# The Potential Use of Carnosine in Diabetes and Other Afflictions Reported in Long COVID Patients

**DOI:** 10.3389/fnins.2022.898735

**Published:** 2022-06-22

**Authors:** Fabiola Cardoso Diniz, Alan Roger Hipkiss, Gustavo Costa Ferreira

**Affiliations:** ^1^Laboratório de Erros Inatos do Metabolismo, Programa de Bioquímica e Biofísica Celular, Instituto de Bioquímica Médica Leopoldo de Meis, Universidade Federal do Rio de Janeiro, Rio de Janeiro, Brazil; ^2^Programa de Pós-Graduação em Ciências Biológicas - Biofísica, Instituto de Biofísica Carlos Chagas Filho, Universidade Federal do Rio de Janeiro, Rio de Janeiro, Brazil; ^3^Department of Pediatrics, Tulane University School of Medicine, New Orleans, LA, United States; ^4^Aston Research Centre for Healthy Ageing, Aston University, Birmingham, United Kingdom; ^5^Programa de Pós-Graduação em Química Biológica, Instituto de Bioquímica Médica Leopoldo de Meis, Universidade Federal do Rio de Janeiro, Rio de Janeiro, Brazil

**Keywords:** carnosine, diabetes, long COVID, SARS-CoV-2 infection, anti-glycating agent

## Abstract

Carnosine is a dipeptide expressed in both the central nervous system and periphery. Several biological functions have been attributed to carnosine, including as an anti-inflammatory and antioxidant agent, and as a modulator of mitochondrial metabolism. Some of these mechanisms have been implicated in the pathophysiology of coronavirus disease-2019 (COVID-19). COVID-19 is caused by severe acute respiratory syndrome-coronavirus 2 (SARS-CoV-2). The clinical manifestation and recovery time for COVID-19 are variable. Some patients are severely affected by SARS-CoV-2 infection and may experience respiratory failure, thromboembolic disease, neurological symptoms, kidney damage, acute pancreatitis, and even death. COVID-19 patients with comorbidities, including diabetes, are at higher risk of death. Mechanisms underlying the dysfunction of the afflicted organs in COVID-19 patients have been discussed, the most common being the so-called cytokine storm. Given the biological effects attributed to carnosine, adjuvant therapy with this dipeptide could be considered as supportive treatment in patients with either COVID-19 or long COVID.

## Introduction

In 2019 the coronavirus disease-2019 (COVID-19) emerged in China (Huang et al., 2020). COVID-19 is caused by severe acute respiratory syndrome-coronavirus 2 (SARS-CoV-2; Al-Kuraishy and Al-Gareeb, 2020). SARS-CoV-2 has quickly spread around the world with devastating consequences (Bedford et al., 2020). The clinical manifestation and recovery time for COVID-19 are variable (Wen et al., 2020; Wiersinga et al., 2020). Most infected patients remain asymptomatic or present mild symptoms, including a flu-like condition consisting of nasal congestion, loss of taste and smell, fatigue, and fever (Wiersinga et al., 2020; Zhou et al., 2020). However, some patients are more severely affected by SARS-CoV-2 infection and may experience respiratory failure, thromboembolic disease, neurological symptoms, kidney damage, acute pancreatitis, and even death (Piroth et al., 2020; Wiersinga et al., 2020; Ye et al., 2020). Mechanisms underlying the dysfunction of the afflicted organs in COVID-19 patients have been discussed, the most common being the so-called cytokine storm include elevated levels of tumor necrosis factor-alpha (TNF-α) and interleukin 6 (Al-Kuraishy and Al-Gareeb, 2020). At the time of discharge from intensive care units, cytokines levels of COVID-19 patients have normally returned to the physiological range (Rodriguez et al., 2020; Wen et al., 2020). Researchers and health professionals are under pressure to reuse, identify and develop new drugs for this global emergency (Sahebnasagh et al., 2020). Based on the biological effects attributed to carnosine, it is suggested this dipeptide can be considered a palliative therapeutic with respect to COVID-19 and long COVID.

## Long-Term Complications

The number of patients recovered from SARS-CoV-2 infection with diseases associated with long COVID is unprecedented and unpredictable (Rando et al., 2021). Long COVID symptoms include physical pain, fatigue, dyspnea, gastrointestinal symptoms, headaches, and memory and psychological disturbances (Mahase, 2020; Mandal et al., 2020; Yelin et al., 2020). These symptoms can evolve over weeks/months following SARS-CoV-2 infection (Mahase, 2020). Whilst some patients report residual COVID-19 symptoms, others may develop new symptoms or new diseases (such as diabetes) long after the initial infection (Cirulli et al., 2020; Korompoki et al., 2021).

## Carnosine and Its Metabolism

Carnosine is a dipeptide composed of β-alanine and L-histidine (Boldyrev et al., 2013). It is expressed in both the central nervous system (CNS) and periphery including skeletal muscle (Nagai and Suda, 1998; Bonfanti et al., 1999; Boldyrev et al., 2013). Carnosine is synthesized by carnosine synthase [EC 6.3.2.11], and its hydrolysis is catalyzed by serum carnosinase (CN1) [EC 3.4.13.20] and/or cytosolic carnosinase (CN2) [EC 3.4.13.3] (Lenney et al., 1982; Lenney, 1990; Teufel et al., 2003). Effective transport of this dipeptide in different cell types occurs *via* peptide transporter 2 (PepT 2; Xiang et al., 2006; Lopachev et al., 2021).

## Biological Actions of Carnosine

Recent studies have revealed that carnosine is present in human erythrocytes and that acetyl-carnosine (resistant to serum carnosine attack) is present in human serum, the concentration of each decrease with age (Chaleckis et al., 2016). Other studies have revealed that very low levels of acetyl-carnosine are strongly associated with human frailty (Kameda et al., 2020), and the blood of patient suffering from age-related macular degeneration contained very low amounts of carnosine (Chao de la Barca et al., 2020). So far, several biological functions have been attributed to carnosine, including as an anti-inflammatory (Fresta et al., 2020) and antioxidant (Jain et al., 2020) agent, and as a modulator of mitochondrial metabolism (Macarini et al., 2014; Shen et al., 2014; Macedo et al., 2016). Supported by the myriad of effects reported, carnosine has been suggested to decelerate aging symptoms (Hipkiss and Brownson, 2000), as well as for the treatment of other diseases, including cardiovascular disease (Menon et al., 2021), neurodegenerative diseases (Caruso et al., 2019), and diabetes (Houjeghani et al., 2018). The latter is mainly supported by its hypoglycemic (Barca et al., 2018) and anti-glycation effects (Pepper et al., 2010; Chilukuri et al., 2018). Additionally, carnosine’s ability to partially suppress glycolysis in a variety of cell types from yeast to tumor cells (Renner et al., 2010; Horii et al., 2012; Hipkiss and Gaunitz, 2014) [perhaps by altering mRNA translation (Son et al., 2008)] may also help to explain the dipeptide’s beneficial effects toward SARS-Cov-2 viral infectivity (Hipkiss, 2020). Indeed, such infection usually induces an upregulation of glycolysis in the infected tissue (Bojkova et al., 2020). While carnosine might exert therapeutic activity toward COVID-19 virus infection (Lopachev et al., 2020; Saadah et al., 2020; Feehan et al., 2021), the possibility that the dipeptide could be protective toward long COVID has not been thoroughly examined.

## Carnosine and Diabetes

The possible application of carnosine for the treatment of diabetes mellitus has been previously discussed (Hipkiss, 2017). Carnosine supplementation mitigates the elevation of glucose, triglycerides, and TNF-α levels in patients with type-2 diabetes (Houjeghani et al., 2018), and/or in overweight or obese pre-diabetic patients (Liu et al., 2015; de Courten et al., 2016). In this scenario, carnosine was shown to suppress glycolysis in different cell types (Hipkiss, 2011). Carnosine also enhances the clearance of a variety of deleterious aldehydes, such as formaldehyde, methylglyoxal and the glycolytic intermediates dihydroxyacetone phosphate and glyceraldehyde-3-phosphate. All of these reactive aldehydes can modify (glycate) proteins, including mitochondrial proteins (Colzani et al., 2016; Hipkiss et al., 2016; Hipkiss, 2017). Methylglyoxal is responsible for many macromolecular modifications associated with secondary complications of type-2 diabetes, for instance enhanced protein glycation (Hipkiss et al., 2016).

## Diabetic Patients at Increased Risk of Worse Coronavirus Disease-2019 Symptoms

Coronavirus disease-2019 patients with comorbidities are at high risk of death (Piroth et al., 2020). The main complicating conditions include hypertension, cardiovascular disease, obesity, chronic obstructive pulmonary disease, and diabetes (Hendren et al., 2020; Wu and McGoogan, 2020). Diabetes is linked to metabolic and macro/microvascular complications that increase morbidity and mortality in different viral infections (Rasheed et al., 2019). SARS-CoV-2 entry into the target cell is facilitated by the connection from the spike protein to a cellular receptor, attaching the virus to the surface of infected cells (Hoffmann et al., 2020). Cellular receptors known to be involved in SARS-CoV-2 infection are the angiotensin-converting enzyme 2 (ACE2) and the transmembrane serine protease (TMPRSS2 and TMPRSS4; Zhang et al., 2003; Hoffmann et al., 2020). Multiple organs are susceptible to SARS-CoV-2 infection, such as the lungs, digestive tract, kidneys, heart, brain, and pancreas (Gavriatopoulou et al., 2020; Puelles et al., 2020). ACE2 is upregulated in patients with cardiovascular disease, hypertension, and diabetes (Pollard et al., 2020). Diabetic patients are at higher risk for the cytokine storm secondary to the pro-inflammatory state triggered by COVID-19 (Apicella et al., 2020), as well as of diabetic ketoacidosis and mortality (Lim et al., 2020). The cytokine storm also leads to peripheral insulin resistance (Kameda et al., 2020) and disrupts pancreatic β-cells functioning, inhibiting insulin secretion (Mehta et al., 2020). The pancreatic damage and hyperglycemia are further stimulated by the direct invasion of SARS-CoV-2 to the pancreas (Zhang et al., 2003). Taken together, these effects contribute to the development of hyperglycemia in COVID-19 patients (Mehta et al., 2020) and potentially induce type 1 diabetes during the long COVID period (Lim et al., 2020). While we strive to understand how COVID-19 induces diabetes or aggravates the existing disease, it is mandatory to maintain long-term follow-up of these patients.

## Recovered Patients at Increased Risk of Developing Diabetes

The term “long COVID” refers to patients with a post-acute COVID-19 (defined as the presence of symptoms 3+ weeks from the onset of symptoms) or chronic COVID-19 (symptoms 12+ weeks; Rubin, 2020; Korompoki et al., 2021). Long-term hyperglycemia in COVID-19 induces oxidative stress, contributing to the development of insulin resistance and dysregulation of pancreatic β-cells (Abdul-Hadi et al., 2020). Many cases of pancreatitis have been reported in COVID-19 patients (Anand et al., 2020; Ghosh et al., 2020; Wang et al., 2020). Pancreatic damage and hyperglycemia may evolve over weeks or months following SARS-CoV-2 infection (Mahase, 2020). In this scenario, reports of late diabetes (and also other complications) in patients recovered from SARS-CoV-2 infection have recently emerged (Mahase, 2020; Morieri et al., 2020; Korompoki et al., 2021).

## Carnosine and Other Coronavirus Disease-2019 Complications

Carnosine was shown to lower the affinity between ACE2 and the spike protein from SARS-CoV-2 (Saadah et al., 2020). So far, no further actions of carnosine on this mechanism have been reported, nor on the interaction of the virus with host cells, neither on viral lifespan. However, given its antioxidant and anti-inflammatory properties (Fresta et al., 2020; Ooi et al., 2020; Scuto et al., 2020), carnosine may attenuate the cytokine storm in COVID-19 patients. Carnosine could also restrain other COVID-19 complications.

A hypercoagulatory state may contribute to an increased mortality in COVID-19 (Mucha et al., 2020). This dysregulated coagulation can be induced by methylglyoxal, which causes post-synthetic protein glycation in diabetics, including the glycation of the anticoagulants anti-thrombin III (Jacobson et al., 2014) and plasminogen (Gugliucci, 2003). Carnosine can inhibit protein glycation and possibly eliminate methylglyoxal, thereby suppressing the anticoagulant modification induced by this reactive carbonyl (Hipkiss, 2020).

The elderly individuals over 50 years old are particularly susceptible to worse complications (e.g., severe pneumonia) and death following coronavirus infections (Jartti et al., 2011), which is also true for COVID-19 (Daoust, 2020). The immune system is impaired during the aging process, rendering the elderly more susceptible to SARS-CoV-2 infection (Wu and McGoogan, 2020). In this context, carnosine’s putative anti-aging properties delayed senescence, lifespan extension, and rejuvenation of cultured human and rodent cells (McFarland and Holliday, 1994; Gallant et al., 2000; Yuneva et al., 2002; Stvolinsky et al., 2010; Boldyrev et al., 2013; Hipkiss et al., 2016) suggest that the dipeptide could be explored. Cellular aging and cell senescence are associated with telomere shortening (Ishikawa et al., 2016) and increases of transforming growth factor-β (TGF-β) signaling and Smad3 expression (Doyle et al., 2010; Baugé et al., 2013). In cultured human cells, carnosine slows telomeres shortening (Shao et al., 2004) and suppresses TGF-β production and signaling, possibly involving inhibition of the Smad2/3 pathway (Köppel et al., 2011). Carnosine can up-regulate coenzyme Q10 synthesis (Schwank-Xu et al., 2021), thereby stimulating mitochondrial activity and contributing to a less aged cellular physiological state. Since CN1 expression increases throughout development (Lenney et al., 1982), a progressive decline in serum carnosine concentrations is observed in elderly humans (Stuerenburg and Kunze, 1999; Tallon et al., 2007) and a correlation between increased CN1 expression and decreased carnosine levels in specific rat brain regions has been detected (Balion et al., 2007). To date, there is no report indicating alterations on carnosine metabolism enzymes elicited by SARS-CoV-2. The brain is an important organ in the clinical presentation of COVID-19. Long COVID patients may also present long lasting neurological symptoms, including the brain fog, hallucinations, double vision, numbness in their limbs or face, disorientation and difficulty concentrating. These symptoms may persist even after 5 months of SARS-CoV-2 infection (Karuppan et al., 2021). Over the last decades, carnosine has been widely suggested as a contributor to brain health (Hipkiss, 2007; Boldyrev et al., 2013; Hipkiss et al., 2016; Kawahara et al., 2018, 2020; Caruso et al., 2019; Schön et al., 2019).

## Administration of Carnosine/Acetyl-Carnosine

Dietary administration of carnosine is hampered by the presence of serum carnosinase in humans. Previous suggestions have included the nasal route especially as this could raise carnosine levels in the olfactory bulb and so perhaps alleviate cognitive impairment, anxiety and long COVID-associated brain fog (Ma and Vervoort, 2020). The presence of the carnosine in the airways may also locally suppress infection. There are no reported studies on the possible efficacy of dietary acetyl-carnosine toward almost any age-related condition, with the exception of lenticular cataracts where the direct application of acetyl-carnosine in solution has been proposed as a therapeutic agent (Boldyrev et al., 2013).

## Discussion

**FIGURE 1:**
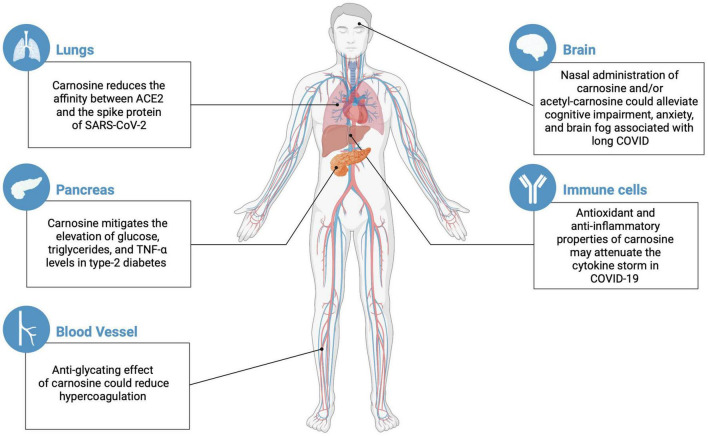
Potential effects of carnosine/acetyl-carnosine on different organs susceptible to COVID and long COVID complications (created with BioRender.com).

The COVID-19 pandemics is a challenging scenario for the global population, researchers, and front-line health professionals (Sahebnasagh et al., 2020). The molecular and cellular bases of COVID-19 are heterogeneous and there is an urge for its complete elucidation. Due to the lack of complete SARS-CoV-2 immunity and of comprehensive vaccination strategy, the unpredictable clinical results of COVID-19 are concerning (Bong et al., 2020; Ma et al., 2020; Ma and Vervoort, 2020). Long COVID is a condition that affects a wide range of patients (Rubin, 2020), which is severely challenging the entire healthcare system. Nevertheless, immunized patients are less likely to develop long COVID compared to the un-immunized (Antonelli et al., 2021). The long-lasting symptomatology can result in chronic morbidity (Shah et al., 2021), including the development of diabetes (Korompoki et al., 2021). The emergence of new COVID-19 variants threatens us with an increase in the proportion of patients suffering from long COVID. Here, we have summarized the available evidence indicating the potential role of carnosine/acetyl-carnosine in ameliorating long-term complications of COVID-19 and diabetes (Figure 1). Whenever possible, the presence of serum acetyl-carnosine and erythrocyte carnosine levels should be measured to determine whether the levels of these peptides could be predictive of morbidity or mortality, and whether raising their levels has any beneficial effects on clinical course and survival. Given the biological effects attributed to carnosine, intranasal adjuvant therapy with this dipeptide could be considered as supportive treatment in patients with either COVID-19 or long COVID.

## Author Contributions

FD and GF contributed to the design, overall collection of information, and writing and final revision of the manuscript. AH was involved in the data collection for diabetes and other long COVID complications, writing and final revision of the manuscript. All authors contributed to the article and approved the submitted version.

## Conflict of Interest

The authors declare that the research was conducted in the absence of any commercial or financial relationships that could be construed as a potential conflict of interest.

## Publisher’s Note

All claims expressed in this article are solely those of the authors and do not necessarily represent those of their affiliated organizations, or those of the publisher, the editors and the reviewers. Any product that may be evaluated in this article, or claim that may be made by its manufacturer, is not guaranteed or endorsed by the publisher.
